# The “adult inactivity triad” in patients with chronic kidney disease: A review

**DOI:** 10.3389/fmed.2023.1160450

**Published:** 2023-03-30

**Authors:** Fan Zhang, Xiaojing Yin, Liuyan Huang, Huachun Zhang

**Affiliations:** ^1^Department of Nephrology, Longhua Hospital, Shanghai University of Traditional Chinese Medicine, Shanghai, China; ^2^Department of Nursing, Longhua Hospital, Shanghai University of Traditional Chinese Medicine, Shanghai, China

**Keywords:** chronic kidney disease, physical activity, sarcopenia, adult inactivity triad, review

## Abstract

**Background:**

The “pediatric inactivity triad” framework consists of three complex, interrelated conditions influencing physical inactivity and associated health risks. Evidence on the beneficial effects of physical activity in adults with chronic kidney disease (CKD) continues to grow, but few studies have explored the complex interactions behind inactivity in this population.

**Results:**

Based on the “pediatric inactivity triad” framework and prior research, we would like to propose a new concept, the “adult inactivity triad” in CKD, including (1) exercise deficit disorder, (2) sarcopenia, and (3) physical illiteracy. Individuals can shift from “adult inactivity triad” to “adult activity triad” and move at different rates and directions along the arrows in each of the three components.

**Conclusion:**

This review explores and summarizes previous research on the three main adult inactivity triad components in the chronic kidney disease population.

## 1. Introduction

Physical inactivity is associated with increased chronic non-communicable diseases, reduced health-related quality of life, and heavy medical expenditures (1–3). The World Health Organization (WHO) has identified physical inactivity as the fourth leading cause of death worldwide (4). Thus, physical activity is associated with primary prevention in the general population and secondary and tertiary protection in patient populations (5). Over 80% of adolescents and 27% of adults reportedly do not meet the WHO-recommended physical activity levels of at least 150 min moderate intensity or 75 min of vigorous-intensity physical activity weekly (6). Since the COVID-19 pandemic, these numbers have become even worse due to the consequences of social isolation, which affects outdoor exercise (7).

The “pediatric inactivity triad,” a condition observed in inactive adolescents and proposed initially by Faigenbaum et al. (8), involves three distinct but interrelated factors that contribute to inactivity: (1) exercise deficit disorder, (2) pediatric dynapenia, and (3) physical illiteracy. Each of these components is important when understood in isolation, but they constitute a “triple jeopardy” (9), which leads to a vicious cycle of decreased physical function and reduced physical activity. This triad not only reminds pediatricians of the complex interactions caused by physical inactivity, but also allows them to recognize the importance of promoting regular physical activity in children (8).

**FIGURE 1:**
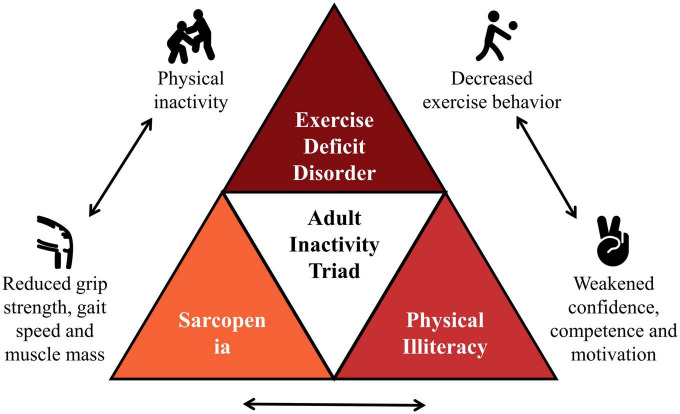
The “adult inactivity triad” in chronic kidney disease (CKD). Adapted from Wilkinson et al. (14).

Chronic kidney disease (CKD) is a major health problem that affects approximately 11.6% in China and 12.9% in the USA, respectively (10). As with most non-communicable diseases, adults with CKD are severely physically inactive, and low physical activity levels are associated with decreased renal function, increased readmission, and all-cause mortality (11–13). Evidence on the beneficial effects of physical activity in adults with CKD continues to grow, but few studies have explored the complex interactions behind inactivity in this population (14). For this reason, building on the results of the pediatric inactivity triad as well as previous studies, we would like to propose a new concept, the “adult inactivity triad” in CKD, including (1) exercise deficit disorder; (2) sarcopenia; and (3) physical illiteracy (Figure 1) (see Table 1 for relevant definitions).

**TABLE 1:** Definitions of related terms.

Terms	Definitions
Physical activity	Physical activity is bodily movement produced by skeletal muscles that result in energy expenditure (15).
Exercise	Exercise is a physical activity that is planned, structured, and repetitive, aimed at improving or maintaining physical fitness (15).
Sedentary behavior	Sedentary behavior is any waking behavior while seated, reclining, or lying posture at <1.5 METs (16).
Exercise deficit disorder	Exercise deficit disorder is described as a condition of reduced levels of moderate to vigorous physical activity (<60 min of moderate to vigorous physical activity daily) that are below recommendations consistent with positive health outcomes (17).
Sarcopenia	Sarcopenia is defined as progressive loss of muscle mass and strength with a risk of adverse outcomes such as disability, poor quality of life, and death (18).
Physical illiteracy	Physical illiteracy refers to the lack of confidence, competence, and motivation to engage in meaningful physical activities with interest and enthusiasm (19).

Given the adverse physical, psychological, and social effects of CKD, a population that tends to lead a sedentary lifestyle, further understanding what factors impact these components is essential to influence change and promptly initiate appropriate interventions.

To ensure the most appropriate literature was reviewed, we systematically searched PubMed and Embase databases for literature related to the adult CKD population with adult inactivity triad components. Patients with the full-spectrum CKD, including non-dialysis, peritoneal dialysis, hemodialysis, and kidney transplantation, were included. There were no restrictions on the study design. Search terms covered include “chronic kidney disease,” “end-stage renal disease,” “physical activity,” “daily steps,” and “sarcopenia.”

## 2. Exercise deficit disorder

Exercise deficit disorder is defined as a condition characterized by lower than recommended levels of physical activity (17). Patients with CKD generally engage in low levels of physical activity or have a sedentary lifestyle for long periods, influenced by cardiorespiratory fitness and muscle health (5). A study by Johansen et al. (20) of 1,547 new dialysis patients from the United States Renal Data System Comprehensive Dialysis Study used the Human Activity Profile revealed a low level of physical activity status [Participants met the low physical activity criterion if they scored in the lowest quintile of the normative data stratified by age and sex on the Adjusted Activity Score of the Human Activity Profile (21)] in the dialysis-dependent CKD population, which was more severe in older and female patients. And then, Beddhu et al. (22) surveyed participants with CKD in National Health and Nutrition Examination Survey, and the data showed that 29.0 and 30.8% were inactive (no reported leisure time physical activity) and insufficiently active (not inactive and did not meet the criteria for recommended levels of physical activity), respectively. Recently Bruinius et al. (23) reported that 3,926 participants in the Chronic Renal Insufficiency Cohort (a study of adults with mild to moderate CKD) study, using time-updated self-reported physical activity to characterize time spent in various physical activities during a typical week over the past month, and about 50% overall also met physical activity guidelines; within this, compared to the lowest moderate to vigorous physical activity quartile, those in the highest quartile were younger, male, did not have prevalent cardiovascular disease, and had an estimated glomerular filtration rate was higher. It is essential to consider that population-based physical activity surveys are primarily questionnaires or self-reports, subject to recall error, and the actual situation may be lower than the reported results.

The traditional measurement of physical activity based on self-reported exercise patterns incurs bias to some extent. A few studies have attempted to link objective data with more subjective measures of physical activity, with mixed results. Results from 110 pre-dialysis CKD patients who wore accelerometers for seven consecutive days to assess their physical activity levels by West et al. (24) showed that this group was sedentary for approximately 79% of day (i.e., mean duration of inactivity = 1152 ± 100 min per day). As assessed by an accelerometer, lower physical activity was reported in hemodialysis-dependent CKD patients compared to sedentary healthy controls, and this difference was more pronounced in older adults (25). Nawab et al. (26) used accelerometer measurements and found that maintenance dialysis patients spent an average of about 1 h (h/day) walking, 0.6 h/day engaging in moderate-intensity activity, 0.7 h/day on light tasks, were only half as active as matched healthy controls, and were sedentary for 13.2 h a day; further analysis found age and leg weakness to be important determinants. An observational analysis of the 2003–2004 National Health and Nutrition Examination Survey by Beddhu et al. (27) found that the mean sedentary time was higher in patients with CKD (40.8 ± 6.8 min/h) than in those without CKD (34.4 ± 7.9 min/h).

### 2.1. Dynamics of physical activity in different stages of CKD

**FIGURE 2:**
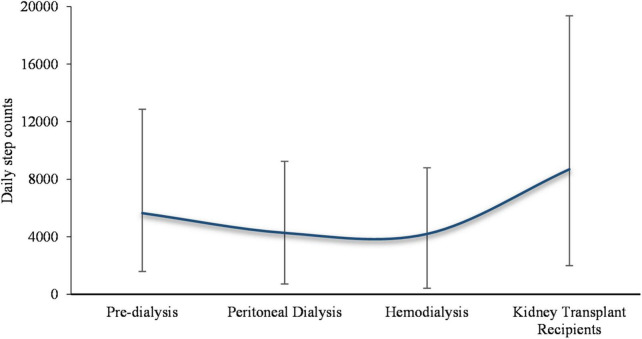
Daily step-based physical activity in patients with different stages of CKD.

Chronic kidney disease is a dynamically progressive non-communicable disease where physical activity deteriorates with disease progression, reaching a nadir at the hemodialysis stage (5). Findings from the Canadian Frailty Observation and Intervention Trial explained this phenomenon, as patients with advanced CKD transition to dialysis experience an accelerated decline in physical function and a subsequent decrease in physical activity (28). A study of 8,444 Canadian patients with early CKD using triaxial accelerometers to measure sedentary time reported that increased sedentary behavior was strongly and independently associated with decreased glomerular filtration rate, with the mean proportion of sedentary time ranging from 58 to 81% (29). Wilkinson et al. (30) using the General Practice Physical Activity Questionnaire in 5,656 patients with various stages of CKD, found that only 6–34% had sufficient physical activity to meet the guideline-recommended amount (30). Our recent meta-analysis quantifying physical activity by daily steps and including 28 observational studies similarly showed that daily step counts in patients with CKD decreased gradually from pre-dialysis to a minimum in hemodialysis-dependent CKD and increased after kidney transplantation (Figure 2) but remained well below the recommended for the healthy population (31).

### 2.2. Physical inactivity/sedentary behavior and poor prognosis in CKD

In recent decades, considerable evidence has highlighted the association of a sedentary lifestyle or physical inactivity with an increased risk of cardiovascular disease and all-cause mortality in patients with CKD. O’Hare et al. (32) first demonstrated an association between sedentary behavior and mortality in a large cohort of dialysis patients, with a 62% increased risk of death over 1 year in sedentary patients compared with non-sedentary dialysis patients (95% CI 1.16 to 2.27). The National Health and Nutrition Examination Survey III-based cohort study of 15,368 adults reported that compared with the inactive group, hazard ratio (HR) in the insufficiently inactive and active groups were 0.58 (95% CI 0.42 to 0.79) and 0.44 (95% CI 0.33 to 0.58) in the CKD population (22). In other words, patients with insufficiently active and active CKD had a 42 and 56% lower mortality risk than the inactive group, respectively. The Dialysis Outcomes and Practice Patterns Study also confirmed that although there may be a benefit to exercising once a week, the risk of death decreases with increasing frequency, meaning that the relationship between the amount of exercise and survival may be a dose-response relationship. Lower mortality rates were observed in participants who exercised daily and more frequently, suggesting that any (at least weekly) exercise is better than no exercise at all (33, 34). A recent study investigating a median of 3.8 years in a hemodialysis population of 6,147 participants reported a 19% (HR: 0.81; 95% CI 0.72 to 0.92) and 21% (HR: 0.79; 95% CI 0.66 to 0.95) lower risk of all-cause mortality and cardiovascular mortality, respectively, in hemodialysis patients with irregular to once-weekly physical activity compared to those without physical activity, while 23% (HR: 0.77; 95% CI 0.65 to 0.91) and 32% (HR: 0.81; 95% CI 0.53 to 0.87) reductions in patients who had physical activity more than once a week to once a day (35).

In patients with full-spectrum CKD, the risk of cardiovascular disease is much higher than in the general population and is the leading cause of death (36), and the association of physical inactivity with cardiovascular events has been demonstrated. Results from a cohort study of 540 kidney transplant recipients followed for 5.3 years showed that an increase in daily physical activity time reduced the risk of cardiovascular events by 38% (HR: 0.62; 95% CI 0.45 to 0.86) after adjusting for confounders (37). In patients with hemodialysis-dependent CKD, each 1000 increase in daily steps was independently significantly associated with cardiovascular events (HR: 0.78; 95% CI 0.72 to 0.84) (38). Similar results were observed in another study of self-reported physical activity (35). In a randomized controlled trial, the CYCLE-HD study showed that 6 months of regular exercise was associated with decreased left ventricular mass and was associated with beneficial left ventricular remodeling. Patients in the regular exercise group had reduced myocardial inflammation and fibrosis compared to the non-regular exercise group (39), both of which are important contributors to arrhythmias and sudden cardiac death. In a review, Bishop et al. (40) concluded that regular exercise improves endothedial nitric oxide release and bioavailability, thereby improving vasodilatation, lowering blood pressure, and improving left ventricular hypertrophy. In conclusion, physical activity is a non-pharmacological intervention for the prevention of cardiovascular events in patients with CKD.

With increased awareness of sedentary behavior, it was realized that simply meeting physical activity levels in public health guidelines is not sufficient, as prolonged sitting may occur during much of the otherwise waking hours, a situation further amplified by the prevalence of COVID-19 (41) and no exception for patients with CKD. Given the high prevalence and lethality of sedentary behavior in CKD patients, Lyden et al. (42) demonstrated the feasibility and effectiveness of a “sitting less and move more” (SLIMM) approach focused on the CKD population. This trial compared an intervention centered on encouraging participants to sit less and move more with standard care, which encourages patients with stage 2–5 CKD to achieve moderate physical activity of 150 min/week, with data collected by the accelerometer at 8, 16, and 24 weeks. The SLIMM group increased daily steps and reduced sedentary behavior at 20 weeks, while no significant changes were observed in the standard care group. Nevertheless, the difference between the SLIMM and usual care groups was significantly attenuated at 24 weeks, resulting in a decrease in the overall effect of the SLIMM intervention on the primary outcome. Kim and Roshanravan (43) suggest that this study informs future interventions targeting physical activity in patients with CKD, i.e., they should focus on reducing sedentary behavior. A cohort study in Taiwan found that compared with patients who were consistently highly active, patients with CKD who changed from highly active to less active were at risk for the composite endpoints of all-cause mortality and end-stage renal disease (HR: 1.39, 95% CI 1.06 to 1.82), all-cause mortality (HR: 2.20, 95% CI 1.55 to 3.13) and increased risk of major adverse cardiovascular events (HR: 2.04, 95% CI 1.32 to 3.14) (13). However, this study included only pre-dialysis patients. The impact of changes in physical activity on the risk of cardiovascular events and death in a dialysis population with a higher incidence of cardiovascular disease and more pronounced sedentary behavior still needs to be validated.

## 3. Sarcopenia

Sarcopenia is prevalent in the CKD population and is characterized by a loss of muscle mass and function (44). Sarcopenia is considered a significant complication in dialysis-dependent CKD patients, along with frailty (45), which is defined as a multisystem impairment associated with increased vulnerability to stress (46). There is much overlap between these two conditions, particularly in the triple-low phenomenon of grip strength, gait speed, and muscle mass, which lead to limited physical function (47, 48).

### 3.1. Prevalence of sarcopenia in CKD

Due to inadequate nutritional intake, inflammation, metabolic acidosis, and dialysis treatment, there is a slight but persistent imbalance between protein synthesis and degradation in patients with CKD, allowing loss of muscle mass to begin in the early stages of CKD, resulting in muscle wasting and decreased physical function with often low physical activity levels (49). As renal function progressively deteriorates, physical activity decreases further during the dialysis treatment phase and often does not return to the pre-dialysis CKD diagnosis or even the pre-dialysis CKD phase, where patients develop significant sarcopenia (5). A recent meta-analysis that included 30 studies showed that the prevalence of sarcopenia in patients with CKD was 28.5% (95% CI 22.9% to 34.1%), with the prevalence varying depending on the population assessed, the method of assessment, the operational definition, and the disease stage (50).

### 3.2. The relationship between sarcopenia and CKD prognosis

Sarcopenia is more prevalent in dialysis-dependent CKD patients but is strongly associated with poor outcomes across the full spectrum of CKD.

#### 3.2.1. Hospitalization

Giglio et al. (51) showed that sarcopenia was associated with a 2.07-fold (95% CI: 1.48–2.88) higher number of hospitalizations in 170 hemodialysis-dependent CKD patients from six dialysis centers followed for 3 years, independent of age, gender, dialysis vintage and diabetes mellitus. For kidney transplant recipients, Chan et al. (52) did not find an independent association between sarcopenia and the composite outcome of mortality and hospitalization followed for 64 months more than 1 year after transplantation. However, when focusing on the sub-dimension of sarcopenia, the authors found that hyposmia was associated with mortality and hospitalization (HR: 2.45). Recent results from de Luca Corrêa et al. (53) on 247 patients with end-stage renal disease followed for 5 years found that hospitalizations were higher in those with sarcopenia (93.8% vs. 49.5%). The findings of sarcopenia and hospitalization rates are limited by the small sample size and do not accurately reflect the attributes of the CKD sample.

#### 3.2.2. Mortality

Sarcopenia is a crucial predictor of increased mortality in patients with CKD. A meta-analysis by Santana Gomes et al. (54) that included three studies reported that sarcopenia increased mortality in non-dialysis CKD patients by 143% (HR: 2.43; 95% CI 1.64 to 3.60). However, the small number of eligible articles included in this meta-analysis limits the interpretability of the results. At the same time, a pooled analysis of eight studies by Ribeiro et al. (55) similarly found that confirmed sarcopenia was associated with an increased risk of death (HR: 1.87; 95% CI 1.35 to 2.59) in patients with dialysis-dependent CKD. Similar results were obtained by Shu et al. (50). Studies on the prediction of sarcopenia on the risk of death in renal transplant recipients are limited. Increases in muscle mass and strength over and above increases in fat mass 2 years after kidney transplantation have been reported to reduce the incidence of sarcopenia in kidney transplant recipients to some extent (56).

#### 3.2.3. Graft outcomes

Although muscle function (including muscle strength and mass) improves in patients with the end-stage renal disease after kidney transplantation, the long-term benefits of graft are not guaranteed. Druckmann et al. (57) retrospectively analyzed data from 183 kidney transplant recipients with sarcopenia assessed by measuring the cross-sectional area and mean muscle density of the psoas major at the level of the third and fourth lumbar vertebrae and the paravertebral muscles at the level of the 12th thoracic vertebrae correlated with poor short-term (postoperative hospital stay) and long-term (risk of death) prognosis after kidney transplantation.

## 4. Physical illiteracy

The third component of the adult inactivity triad is physical illiteracy, which refers to a lack of confidence, ability, and motivation to engage in meaningful physical activity with interest and enthusiasm (58). Physical illiteracy includes learning in the psychomotor, cognitive, and affective domains; therefore, interventions need to be augmented with effective cognitive strategies to enable inactive middle-aged and older adults with CKD to learn the value of the physical activity.

Exercise self-efficacy is the confidence people have in their ability to exercise and is an important and modifiable predictor of physical activity and exercise behavior (59). Self-efficacy and exercise behavior are interrelated, with exercise self-efficacy increasing through mastery experience as individuals gain exercise experience, while self-efficacy is also a catalyst for maintaining motivation to exercise (60, 61). As self-efficacy increases, a person tends to exercise more often, and this idea has also been validated in kidney transplant recipients. Hu et al. (62) elaborated from the perspective of the Health Action Process Approach (HAPA) model that planning and recovery self-efficacy were significant predictors of physical activity. Thus, nephrology health providers can promote mastery of the experience by setting achievable goals and highlighting patients’ achievements in overcoming exercise barriers. However, a survey showed that 40% of physicians tended to be concerned about the risks associated with physical activity compared to a minority of patients (8%) (63). Lack of motivation is also a barrier to physical activity participation in the CKD population. In the study by Moorman et al. (64) 57% of patients agreed that they would exercise if their physician suggested it.

## 5. Future considerations

**FIGURE 3:**
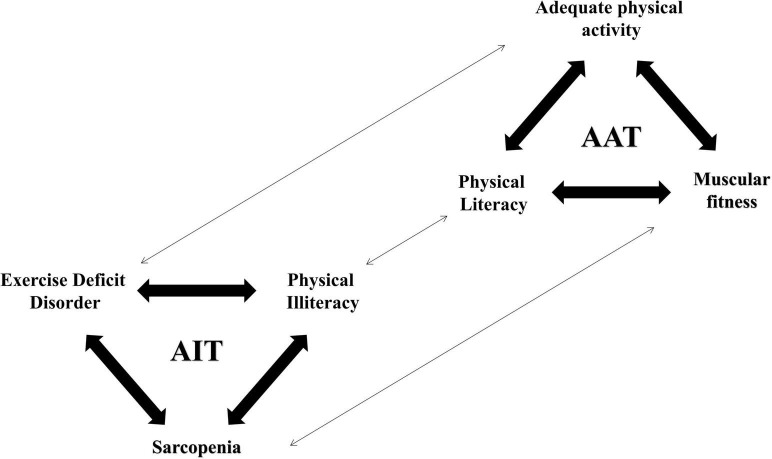
The adult inactivity triad–adult activity triad continuum of physical activity in adult CKD patients. Adapted from Faigenbaum et al. (8).

The opposite of the “adult inactivity triad” is the “adult activity triad” (Figure 3), which has components of adequate physical activity, physical literacy, and muscular fitness (i.e., strength, power, and endurance). Individuals can shift from “adult inactivity triad” to “adult activity triad” and move at different rates and directions along the arrows in each of the three components. In contrast to the “adult inactivity triad,” “adult activity triad” has significant benefits for the health and well-being of individuals with CKD (65). In addition to physical health, increasing and maintaining physical activity levels in CKD patients also benefit psychological and social health. Based on this, research directions for future studies should focus on the following:

(1) Promoting physical activity by changing the behavior of CKD patients using objective tools such as pedometers or accelerometers.
(2) The effects of intervention strategies incorporating nutritional supplementation combined with exercise training on the reversibility of muscle health (sarcopenia and frailty).
(3) Physical literacy as the root cause of physical activity participation in people with CKD, based on self-regulation theory to guide individuals’ thoughts, behaviors, and feelings to change confidence and motivation.

## 6. Conclusion

Physical inactivity and sedentary behavior are influential risk factors for the development of chronic disease, contributing to morbidity and mortality, as well as to the economic burden on society through the provision of health and social care and reduced occupational productivity. Given that most patients with CKD do not accumulate sufficient daily physical activity, innovative strategies are needed to change this situation. This review presents the adult inactivity triad for CKD patients against the pediatric inactivity triad and summarizes the current literature. This framework collects three interrelated factors driving physical inactivity in CKD patients. The literature shows that patients at all stages of CKD face three components of the adult inactivity triad (i.e., exercise deficit disorder, sarcopenia, and physical illiteracy). A concerted effort by nephrologists, cardiologists, and nurses, is needed. If our goal is to prevent further declines in physical activity, further work is needed to determine the best interventions for sedentary lifestyle changes in patients with CKD.

## Author contributions

FZ, XY, and LH collected the data, wrote the draft of the manuscript, and revised by HZ. All authors obtained the consent to publish, contributed to the article, and approved the submitted version.
